# ATM is associated with the prognosis of colorectal cancer: a systematic review

**DOI:** 10.3389/fonc.2025.1470939

**Published:** 2025-03-12

**Authors:** Pei Wu, Zelin Wen

**Affiliations:** ^1^ Department of Gastrointestinal Surgery, Yongchuan Hospital of Chongqing Medical University, Chongqing, China; ^2^ Department of Pancreatic and Gastric Surgery, National Cancer Center/National Clinical Research Center for Cancer/Cancer Hospital, Chinese Academy of Medical Sciences and Peking Union Medical College, Beijing, China

**Keywords:** colorectal cancer, ataxia-telangiectasia mutated (ATM), prognosis, system review, meta-analysis, radiotherapy, chemotherapy, immunotherapy

## Abstract

**Objective:**

Chemosensitivity and radiosensitivity are associated with the prognosis of colorectal cancer, and the expression of the ataxia-telangiectasia mutated (ATM) protein plays an essential role in these processes. The present study examined the relationship between ATM expression and the survival outcomes of colorectal cancer patients and explored the underlying mechanism and promising therapeutic strategies.

**Method:**

A search including medical subject headings (MeSH), free terms, and combined words was conducted using Pubmed, EMBASE, and Cochrane. Studies had to meet the inclusion criteria as well as include processes such as data extraction and quality evaluation. The survival outcomes were assessed using hazard ratio (HR) and 95% confidence interval (CI). Heterogeneity, and publication bias were analyzed, and a P value <0.05 was considered statistically significant.

**Results:**

Nine studies with 2883 patients were included in the meta-analysis. Low ATM expression level was related to poor overall survival (HR=0.542, 95% CI=0.447–0.637; P=0.000). Disease-free, progression-free, and recurrence-free survival rates were lower in patients with low ATM expression than in those with high ATM expression. There was no significant difference between Stage I–II and Stage III–IV colorectal cancer patients [risk ratio (RR)=1.173, 95% CI=0.970–1.417, P=0.690].

**Conclusions:**

Low ATM expression level may be a marker of poor survival in colorectal cancer and contributes to resistance to therapy. Targeting related factors in these pathways to sensitize tumors to treatment is a potential therapeutic strategy, and monitoring ATM status could be a valuable guide independent of the immunotherapy or chemotherapy strategy used.

## 1 Introduction

Colorectal cancer (CRC) is one of the most common cancers worldwide and a leading cause of cancer-related mortality among both men and women (1). In China, CRC ranks fifth as a cause of cancer-related death among all cancers (2). According to the latest epidemiology studies in China, CRC incidence and mortality are increasing compared with the latest Cancer Statistics report in 2015 (3). The treatment of cancer consists primarily of radical surgery combined with adjuvant therapy such as chemotherapy, radiotherapy, targeted therapy, and immunotherapy (4). The development of next generation sequencing led to the identification a variety of mutations, and precision medicine addressed the relationship between clinical treatment and gene alterations, resulting in decreased treatment toxicity, better survival, and improved quality of life of patients (5). Because of the increased incidence and mortality of CRC, identifying therapeutic targets is essential to open the era of precision medicine based on gene expression status.

The protein kinase of ataxia-telangiectasia mutated (ATM), a serine/threonine-protein kinase, is a critical repair factor that is recruited to and activated by double-stranded DNA breaks (DSBs). This is a vital process for maintaining normal cell division, and disruption of this system can promote carcinogenesis and is associated with poor prognosis in cancer (6, 7). Genetic variation in ATM is associated with poor survival in CRC; however, Sundar et al. (8) reported that patients with deficient ATM expression have better survival outcomes because they show increased sensitivity to DNA damage agents such as oxaliplatin (8). This underscores the need to explore the mechanisms underlying the role of ATM expression in CRC.

The most effective adjuvant treatments for patients with CRC are radiotherapy; chemotherapy regimens composed of 5-fluorouracil, oxaliplatin, and irinotecan; targeted agents such as anti-angiogenic compounds (bevacizumab or aflibercept) or anti-epidermal growth factor receptor (EGFR) drugs (cetuximab or panitumumab) according to the RAS/BRAF status of the tumor; and immunotherapy according to mismatch repair (MMR) alterations and microsatellite status (9). ATM expression status can influence the effect of therapy through multiple processes, although the association between ATM status and CRC-related processes has not been systematically reviewed to date. In order to verify the effect of ATM status on the prognosis of CRC patients, we performed a systematic review of the literature. Studies containing data on ATM expression status and survival outcomes were analyzed to explore the effect of ATM on the prognosis of CRC patients. Tumor stages were analyzed further to determine their importance for patient survival. Related studies were summarized to identify underlying mechanisms and pathways affecting survival outcomes.

## 2 Materials and methods

### 2.1 Literature search, data extraction, and quality evaluation

The Pubmed, Cochrane, and Embase databases were searched for related studies. The search included MeSH terms, free terms, and combined words. After the literature search, all studies were screened in Endnote according to the keywords and objectives. The selected studies were subjected to quality evaluation according to the Newcastle–Ottawa Scale (NOS), which is one of the most useful methods for assessing the quality of nonrandomized studies, and only high or medium quality studies were included (**Table 1**). The acquired studies were used for data extraction, which was performed by two authors (ZW and PW) according to a predesigned data extraction form, including the name of the first author, sample size, tumor stage, and survival outcome (**Table 1**).

**Table 1:** Characteristics of all studies.

Study	Year	Stage	ATM expression		Survival outcome	HR	CI	Newcastle–Ottawa Scale			Study quality
			Higher	Lower				Selection	Comparability	Outcome	
***Heike Grabsch*** (10)	2006	I-IV	241	68	OS	0.6	0.38-0.94	★★★☆	★★	★★☆	High
***Sumana Narayanan*** (11)	2019	I-IV	146	137	OS	0.5	0.35-0.58	★★★☆	☆☆	★★☆	Fair
***Yuanfang Lu*** (12)	2014	I-IV	44	68	OS	0.4	0.13-0.82	★★★☆	☆☆	★★☆	Fair
***Raghav Sundar*** (8)	2018	NA	206	17	OS	0.7	0.37-1.33	★★★☆	☆☆	★★☆	Fair
***Giovanni Randon*** (13)	2019	IV	192	35	OS	0.6	0.33-0.98	★★★☆	★★	★★☆	High
***Peng-Chan Lin*** (14)	2021	III	86	22	OS	0.7	0.08-6.57	★★★☆	☆☆	★★☆	Fair
***DM Kweekel*** (15)	2009	NA	63	28	PFS	0.3	0.10-0.94	★★★☆	☆☆	★★☆	Fair
***Jan Dimberg*** (16)	2020	II	21	64	RFS	0.1	0.03-0.53	★★★☆	★★	★★☆	High
***Andrew D Beggs*** (17)	2012	II-III	908	537	DFS	0.6	0.40-0.90	**★★★☆**	**★★**	**★★☆**	High

high quality, 6 positive answers; low quality, 3 positive answers; fair quality, 4 or 5 positive answers.

### 2.2 Inclusion and exclusion criteria

The inclusion criteria were patients pathologically diagnosed primary CRC, data on ATM detection, data on survival outcome acquired directly from studies or extracted from the Kaplan–Meier curve generated with the Engauge Digitizer (http://digitizer.sourceforge.net/), which were used to calculate the hazard ratios (HRs) using the method described by Tierney et al. (18), and studies with a sample size of >30 patients. The Newcastle Ottawa Scale was used to assess the quality of included studies; if the study quality after evaluation of the “selection,” “comparability,” and “outcome” items in the scale was low (positive answers ≤3) or the survival outcome data could not be obtained, the study was excluded.

### 2.3 Endpoints, heterogeneity, and publication bias

The endpoints included overall survival (OS), disease-free survival (DFS), progression-free survival (PFS), and recurrence-free survival (RFS). The hazard ratio (HR) and 95% confidence interval (CI) were used to express survival outcomes; the risk ratio (RR) and 95% CI were used to analyze differences in tumor stage between two groups. Heterogeneity was quantified using the I2 statistic. P <0.05 and I2 >50% were considered substantial heterogeneity, and a random effects model was then used. Publication bias was detected using Egger’s and Begg’s tests, where P <0.1 was regarded as confirmation of significant publication bias.

### 2.4 Statistics

Images were processed with the Engauge Digitizer and Adobe Photoshop CC 2018 (Adobe Inc., San Jose, CA, USA). Survival analyses were performed using STATA 14.0 software (Stata LLC, College Station, TX, USA). A P value <0.05 was regarded as statistically significant.

## 3 Results

### 3.1 Study characteristics and quality

**Figure 1:**
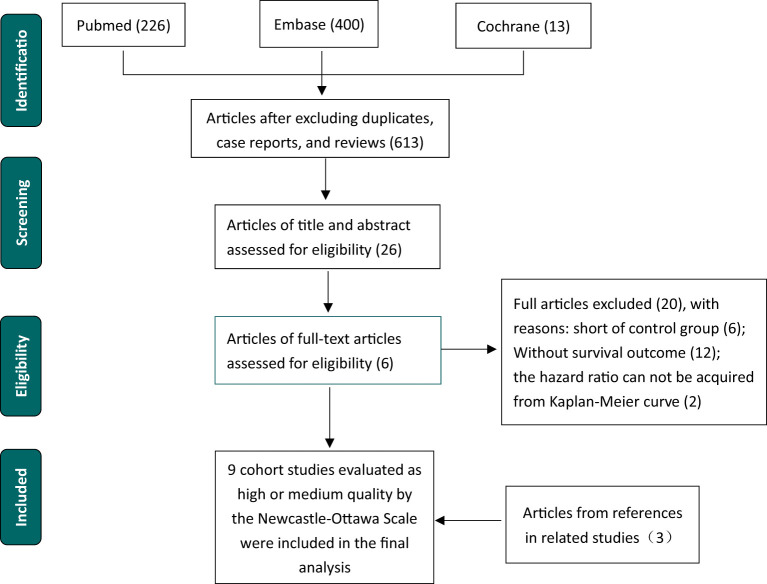
Flow chart of selection procedure literature.

After a literature review, nine studies with 2883 patients were included in the meta-analysis (**Figure 1**). Excluding Sundar et al. (8) and Lin et al. (14), studies supported the association between low ATM expression level and poor survival, including OS, PFS, DFS, and RFS. All cohort studies were evaluated based on the Newcastle–Ottawa Scale (NOS) to be assessed as high or median stages (**Table 1**).

### 3.2 Pooled results

**Figure 2:**
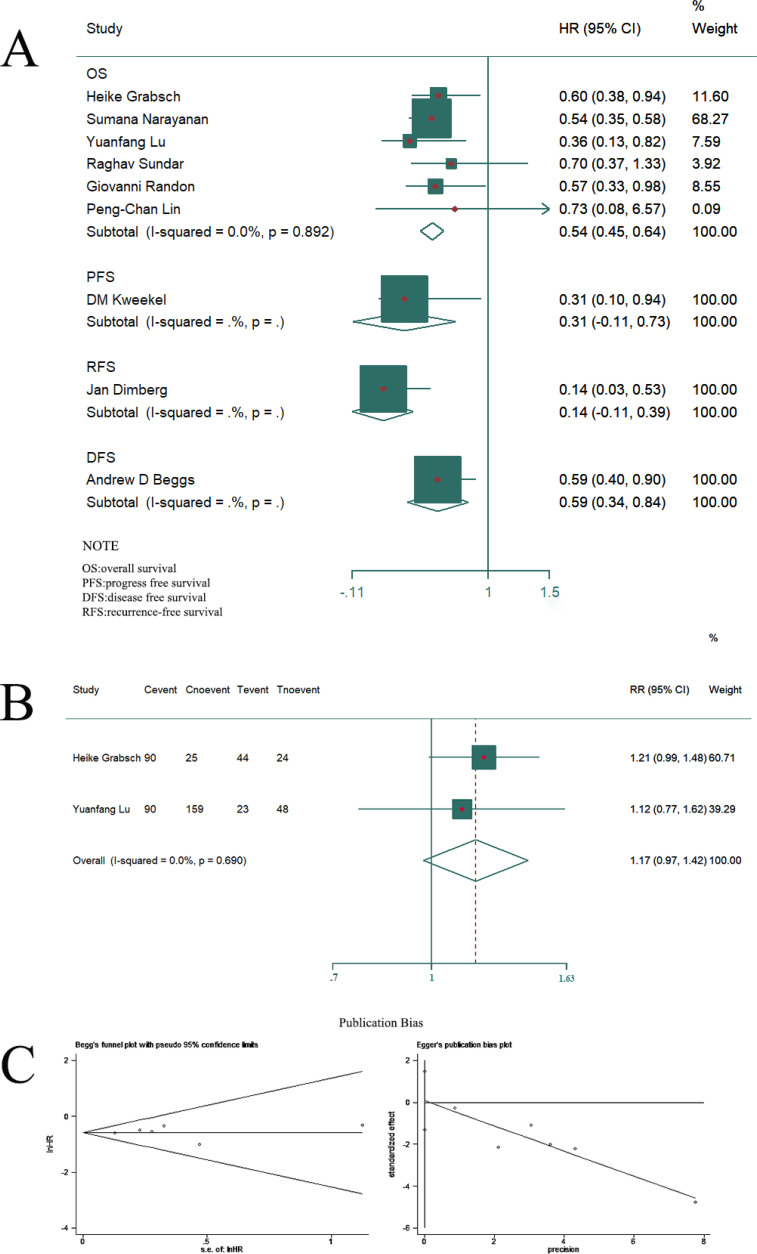
Compared with the low expression group, high expression of ATM was associated with superior survival outcomes, including OS, PFS, DFS, and RFS **(A)**. There was no significant difference in tumor stage between ATM mutation or not **(B)**. There was no publication bias according to Egger’s and Begg’s tests **(C)**.

We pooled the HR for OS according to the initial results, which suggested that low ATM expression is related to poor OS (HR = 0.542, 95% CI = 0.447–0.637; P = 0.000) (**Figure 2A**). DFS, PFS, and RFS were lower in patients with low ATM expression than in those with high expression. Finally, we explored the connection between ATM expression and tumor stage. The results showed no significant difference between Stage I–II and Stage III–IV CRC patients regarding ATM expression (RR = 1.173, 95% CI = 0.970–1.417, P = 0.690) (**Figure 2B**). No heterogeneity or publication bias was detected based on Egger’s test (P> |t| = 0.875) and Begg’s test (Pr > |z| = 0.851) (**Figure 2C**), and I2 (P = 0.892).

In the final result, although we concluded that Low ATM expression level was associated with poor survival outcomes, a systematic review is necessary to explore the relationship between ATM status and treatment strategies because of the shortage of related subgroup results in present studies and already existed promising study results about ATM status in colorectal cancer patients. Subsequently, aiming at the clinical treatment, the effects of ATM status on radiotherapy, Mismatch repair/microsatellite status, and chemotherapy were systematically summarized and provided novel insights for further studies and treatments.

## 4 Discussion

### 4.1 The prognosis of CRC patients

CRC is a highly heterogeneous disease with respect to its clinical and biological features, resulting in striking differences in disease progression and treatment response among patients (19). CRC patients are categorized into four subtypes according to the consensus molecular subtype (CMS 1–4), a thoroughly studied and robust stratification strategy for CRC. ATM mutations are present in 7% of non-hypermutated tumors in patients with the CMS3 subtype and are associated with a poor prognosis and limited treatment options (7, 20). In the present study, we performed a systematic review to summarize the effect of ATM expression level on survival outcomes in CRC patients. Nine studies with 2883 patients were included, and patients were classified into high ATM expression (n = 1907) and low ATM expression (n = 976) groups. Most of the studies analyzed found an association between low ATM expression level and inferior survival in CRC patients except the studies by Sundar et al. (8) and Lin et al. (14) After combining survival outcomes, low ATM expression was associated with poor survival, whereas no significant difference in tumor stage was observed between the low and high ATM expression groups. This result is consistent with the function of ATM in the response to DSBs and poor molecular subtypes. Understanding the relationship between ATM and known prognostic factors may provide insight into the utility of ATM expression level as a potential therapeutic approach. For example, in the studies analyzed, high ATM expression levels in CRC were related to better survival outcomes than low ATM expression levels because of the role of ATM in DSB repair and MMR.

### 4.2 Mismatch repair/microsatellite status

The mismatch repair (MMR) system is mainly composed of four proteins (MLH1, MSH2, MSH6, and PMS2) involved in the repair of base-base mismatch that occurs during DNA replication in proliferating cells, which can lead to the accumulation of mutations that fuel carcinogenesis. Microsatellite instability (MSI) is a molecular marker of MMR deficiency (dMMR) and occurs in approximately 15% of CRCs (21, 22). Microsatellite status is considered as a prognostic indicator and a predictor of the response to immunotherapy against targets such as programmed cell death-1 (PD-1) and programmed death-ligand 1 (PD-L1). Grabsch et al. reported that loss of the MMR proteins MLH1 and MSH2 is related to reduced expression of ATM because of effects on the respective gene loci and is associated with significantly longer overall survival (10). Narayanan et al. reported that low ATM expression is associated with high MSI (MSI-H)/dMMR, which is related to helper T-cells and M1 macrophages, finally leading to improved survival (11). Immune checkpoint blockade with PD1 inhibitors is the standard of care for the first-line treatment of MSI-H/dMMR metastatic CRC; however, MSI-H/dMMR represents a small proportion of CRCs, and immune checkpoint inhibitor therapy is largely ineffective for metastatic microsatellite-stable (MSS)/proficient MMR (pMMR) patients (23). Zhou et al. reported that ATM mutations significantly increase immune activity in MSS colon adenocarcinoma (COAD) patients, supporting the feasibility of using ATM mutation status as a predictor of the immunotherapy response in MSS COAD (24). Alterations in ATM expression often manifest as an ultra-high tumor mutational burden, which is associated with a better response to immunotherapies (25–27). The relationship between ATM mutation and MMR/microsatellite status may provide novel insight into the response to immune therapy in CRC, especially for MSS CRC (27). However, additional clinical studies are necessary to elucidate the underlying mechanisms. The connection between ATM and microsatellite status will be a promising research direction considering its immediate relevance to clinical treatments.

### 4.3 Radiotherapy

**Figure 3:**
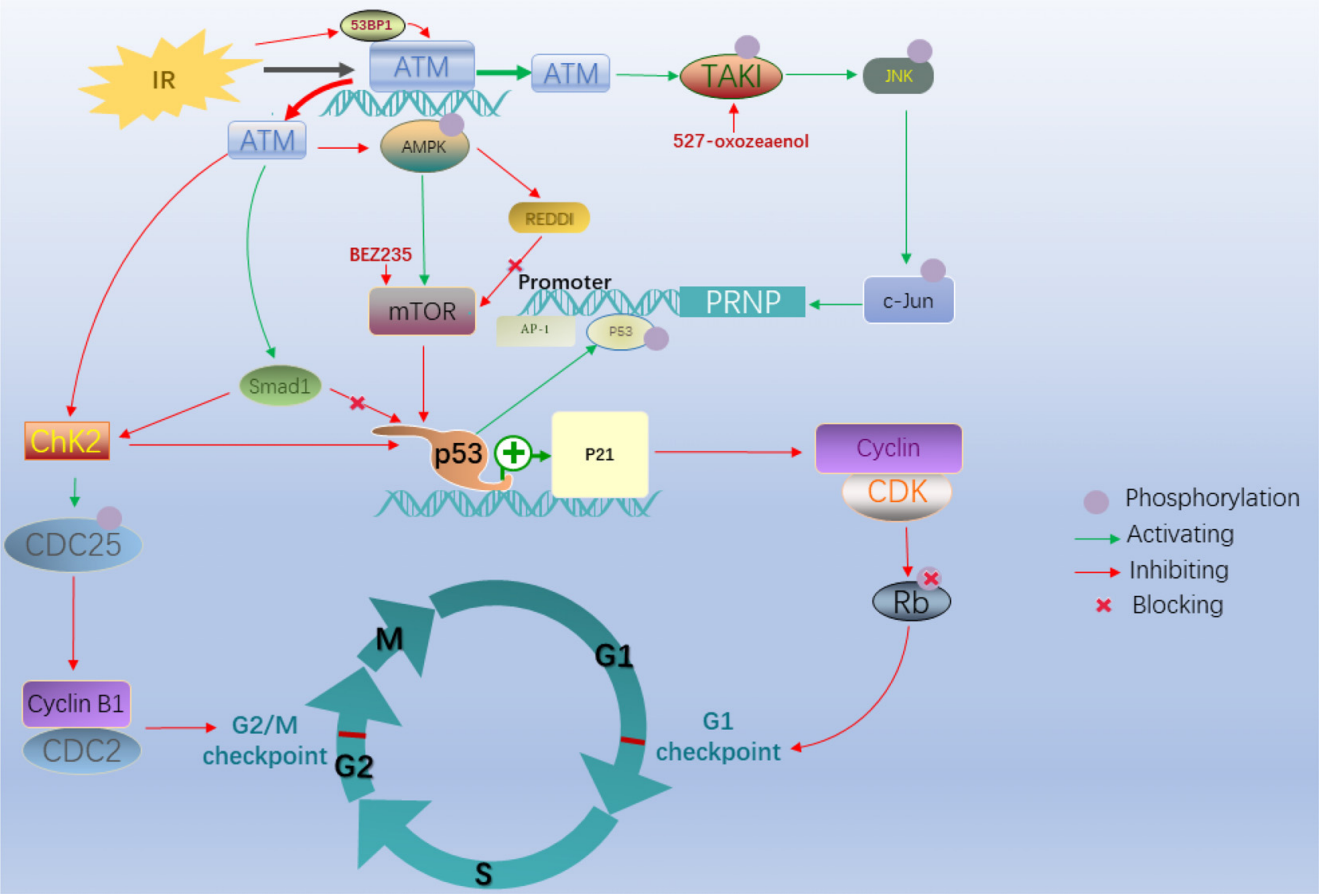
After ionizing radiation, G1/S and G2/M cell cycle arrest plays an essential role in radioresistance. In these processes, the P53-dependent ATM/p53/p21 pathway and ATM–Chk2 pathway with the absence of P53 are major signaling pathways leading to G1/S and G2/M cell cycle arrest, respectively. The ATM-CHK2-p53 pathway is activated when vital factors alternate including degraded CDC25C and deficiency of 53BP1. The ATM-TAK1-PrPC pathway is another essential pathway mediating radioresistance in colorectal cancer cells.

Radiation therapy (RT) is a mainstay of colorectal cancer treatment (CRC treatment), especially for rectal cancer; however, resistance to RT limits the efficacy of treatment, especially in patients with advanced disease. DSB repair plays a vital role in acquired resistance to radiotherapy and chemotherapy in many cancers (28, 29); therefore, it is essential to clarify the involvement of ATM in these processes. When DNA DSBs appear after exposure to factors such as chemotherapy or radiotherapy, ATM is recruited and binds to the location of DSBs to repair the damage; therefore the expression level of ATM thus plays a critical role in this process (30). However, this repair mechanism is also responsible for resistance, leading to poor survival outcomes (31). Given that the relationship between ATM and DBSs is associated with radioresistance, targeting this response represents a promising approach to improving the efficacy of RT (32, 33). This process includes various mechanisms influencing radiosensitivity according to previous studies (34, 35) (**Figure 3**), and cell cycle arrest is a vital alteration among these. Cell cycle checkpoints control various mechanisms in the eukaryotic cell cycle by examining internal and external cues at every stage and determining whether cells move forward with division. These include DNA structure checkpoints (DSCs or DDCs) and spindle assembly checkpoints (SACs) (36). Cells can undergo arrest at G1, S, and G2/M points after ionizing radiation (IR)-induced DNA damage (37). Radiosensitivity is closely associated with cell cycle arrest in these processes (38).

#### 4.3.1 p53 (-/-)

Regarding the mechanisms responsible for the decreased radiosensitizing effects of ATM alteration, cell cycle arrest plays a vital role. The tumor suppressor p53 is critical for these checkpoint pathways (39–41). The different status of p53 determines the distinct ATM pathway that decreases radiosensitivity because of IR-induced DNA damage in CRC cells (39, 42). In the presence of p53 deficiency, the checkpoint arrest primarily relies on the CHK (Checkpoint kinase)–dependent pathway (43). For example, in the ATM–checkpoint kinase 2 (CHK2) pathway, ATM mutation induced by DSBs initially phosphorylates the CHK2 protein kinase encoded by the tumor suppressor gene CHEK2, which is involved in cell cycle arrest, DNA repair, or apoptosis in response to DNA damage (44); subsequently, this active kinase phosphorylates cell division cycle 25C (CDC25C) at Ser216 and inactivates it, which in turn inhibits the activity of the CDC2-cyclin B1 complex and finally triggers G2/M phase arrest. However, if CDC25C is degraded in the IR response, the ATM-CHK2-p53 pathway would replace the above mechanism to monitor G1 checkpoint arrest when p53 is proficient, which is an essential regulatory mechanism used by cells to block mitotic entry in response to DNA damage (45). CHK2 is a traditional target of ATM in the DNA damage response, although the ATR-CHK1 pathway is more common (46). Nevertheless, ATM is also required for CHK1 activation under certain circumstances (47).

#### 4.3.2 p53 (+/+)

Compared with the absence of p53 expression, cells expressing p53 (+/+) are primarily dependent on the ATM/p53/p21 pathway (48, 49). Regarding cell cycle checkpoints, multiple downstream targets of ATM determine the different outcomes. For example, adenosine monophosphate-activated kinase (AMPK), a metabolic sensor, acts as an upstream inhibitor of mTOR activity, which regulates cellular responses to IR (50–52). In this pathway, IR contributes to inhibiting the phosphorylation of both AMPK and its substrate in an ATM-dependent manner; the absence of AMPK stimulates mTOR activity by inhibiting the expression of the mTOR inhibitor REDD1, which not only acts as a classical pathway inducing tolerance to radiotherapy, but is also associated with the canonical p53-dependent G1 phase arrest pathway (50). Overexpression of p53 may cause p21 to accumulate, leading to inhibition of cyclin-CDK complex activity, which prevents the phosphorylation of retinoblastoma protein, which is required for progression to the S phase (53). This process ultimately results in G1/S arrest. However, when ATM upregulates Smad1 in the DNA damage response, ATM–CHK2 is activated after stabilization of p53, resulting in G2/M phase arrest (54). Decreased expression of p53 binding protein 1 downregulates ATM and CHK2, which affects the cell cycle leading to radiotolerance of CRC cells through the ATM-CHK2-p53 pathway (55, 56).

#### 4.3.3 Prion protein

Compared with decreased expression of ATM in many pathways, including the ATM/p53/p21, ATM–CHK2, and ATM-CHK2-p53 pathways, IR could activate ATM to respond to oxidative stress in tumors, resulting in resistance associated with the expression of proteins such as the cellular prion protein (PrPC), which is related to stemness, invasiveness, resistance to chemotherapy, and radiosensitivity (57–60). Increased levels of PrPC contribute to acquired resistance to radiotherapy in CRC. Jacqueline et al. found that the ATM-TAK1-PrPC pathway plays an essential role in mediating radioresistance in CRC cells (61). Exposure of CRC cells to irradiation activates c-Jun, followed by activation of ATM and induction of TAK1-dependent phosphorylation of JNK. An AP-1 binding site in the PRNP promoter results in increased levels of PrPC.

In theory, because the protein kinase activity of ATM mediates the cellular response to DNA damage induced by IR, ATM is involved in inducing radioresistance according to multiple pathways in CRC cells (62). Identifying novel targets and designing drugs according to these processes are promising strategies (63). For example, Sherri et al. demonstrated that 527-oxozeaenol, which acts as a pharmacological inhibitor of TAK1, enhances the radiotherapeutic effect in CRC cells according to the ATM-TAK1-PrPC pathway (61). BEZ235 acts as an effective radiosensitizer of CRC cells and prolongs the radiotherapeutic effects by suppressing the activation of ATM and DNA-PKcs-associated DNA repair (35, 64). Lin et al. reported that quercetin-induced radio-sensitization is mediated by the inhibition of ATM kinase (65). Although the efficacy of ATM inhibitors such as KU-55933, KU-60019, KU-59403, CP-466722, AZ31, AZ32, AZD0156, and AZD1390 has been demonstrated in different tumor types showing different responses to RT in preclinical studies (28, 66–68), systematic and comprehensive testing is lagging in CRC (7). A phase I clinical trial evaluating the safety and tolerability of AZD1390 in combination with RT (ClinicalTrials.gov Identifier: NCT03423628) is currently in the recruiting phase.

### 4.4 Chemotherapy

To the best of our knowledge, in addition to primary alterations and DNA damage induced by radiotherapy (69), chemotherapy-induced DNA alterations remain the underlying cause of ATM alteration. Fluoropyrimidine (5-fluorouracil or capecitabine) as a single agent or in combination with oxaliplatin is the standard first-line chemotherapy for CRC, which consolidates the curative effect and prolongs the survival time in CRC patients (70, 71). However, chemosensitivity and acquired drug resistance after repeated exposure to chemotherapy agents are the leading cause of cancer and even death (32, 72). A series of studies provide evidence that ATM regulates cellular defenses against certain cytotoxic agents (73–75). For example, although the DNA damage drug oxaliplatin acts as an effective chemotherapeutic drug (76, 77), Kweekel et al. reported that patients with mutated ATM have a 4.25-fold higher risk of progression on capecitabine plus oxaliplatin than patients with wild-type ATM (15), and concomitant chemoresistance after treatment limits the therapeutic efficacy in CRC patients (75). Previous studies found that the expression of Bmal1 (78), isocitrate dehydrogenase 2 (IDH2) (79), and miR-203 (80) is associated with resistance to oxaliplatin, and ATM is a crucial factor in these processes (80, 81). Mechanistically, overexpression of the circadian clock gene Bmal1 could increase sensitivity to oxaliplatin by regulating G2–M arrest through the activation of the ATM pathway (78); however, the abrogation of IDH2 could also increase the efficacy of oxaliplatin by promoting phosphorylation of the ATM protein; furthermore, the expression of miR-203 could contribute to oxaliplatin resistance by negatively regulating ATM kinase (80). 5-Fluorouracil (5-FU)-based chemotherapies are essential in the treatment of CRC (82). Morcillo et al. demonstrated that p38MAPK activation plays a role in the resistance to 5-FU, and ATM shows a redundant function in this mechanism (83). However, altered ATM expression is not the only decisive factor for the poor prognosis; epigenetic alteration of ATM, such as aberrant methylation of the ATM promoter, also plays an essential role in the resistance to treatment (34). Genetic variants of ATM also affect the survival outcome. For example, the G allele in ATM rs609429 is associated with longer OS than the C/C variant in refractory metastatic CRC patients receiving Tas-102 chemotherapy (84).

The clinical application of Poly (ADP-ribose) polymerase (PARP) inhibition in CRC has gained attention based on data showing that PARP inhibitors (PARPi) improve prognosis when combined with chemotherapeutic agents such as platinum agents and irinotecan, and ATM deficiency increases sensitivity to PARP inhibition (85). CRC patients with ATM mutation are highly sensitive to PARPi/chemotherapy combination at low doses, regardless of the CMS and microsatellite status. This is attributed to a delay in the resolution of DSBs through homologous recombination repair (HRR), which acts in coordination with the S and G2 checkpoint machinery to eliminate chromosomal breaks before cell division occurs (75). Therefore, ATM status may be a valuable strategy for determining the efficacy of anticancer agents.

### 4.5 Limitations and perspectives

Here, we performed a meta-analysis and showed that low ATM expression level is associated with poor survival and possibly decreased chemosensitivity and radiosensitivity. Although no significant heterogeneity or publication bias was detected in the present study, there were limitations, such as a lack of subgroup and data analysis of specific pathways. However, we systematically reviewed the related mechanisms presented intuitively, including a cell pathway map, explored the effects of ATM alteration on therapeutic strategies in CRC, and examined the promising application of ATM in clinical practice.

Although most of the studies included in the meta-analysis supported that patients with low ATM expression levels have shorter survival times than those with high ATM expression levels, multicenter randomized controlled trials are still needed to confirm these findings. In addition, further cell or animal experiments are necessary to explore the role of the relevant mechanisms in CRC, especially regarding MMR/microsatellite status, radiotherapy, and chemotherapeutic regimes. Finally, some studies suggest that ATM can be used as a marker to guide treatment or as a sensitizer. However, it is essential to conduct multicenter randomized trials to explore the relationship between ATM and chemotherapeutic regimes and screen the definite indications in clinical practice.

## 5 Conclusion

Low-level ATM expression may be a marker of poor survival in CRC and contributes to resistance to therapy, and targeting this mechanism could potentially increase sensitivity to treatments. ATM expression status was identified as a promising marker for guiding immunotherapies and chemotherapies, especially for MSS CRC patients, and for the design of chemotherapeutic regimes such as combination treatments with PARPi.

## Data Availability

The original contributions presented in the study are included in the article/supplementary material. Further inquiries can be directed to the corresponding author.
